# Anesthesia Workspace Cleanliness and Safety: Implementation of a Novel Syringe Bracket Using 3D Printing Techniques

**DOI:** 10.1155/2019/2673781

**Published:** 2019-07-01

**Authors:** Dustin R. Long, Allison Doney, Devan L. Bartels, Crystal E. Tan, Puneet K. Sayal, Thomas A. Anderson, Aalok V. Agarwala

**Affiliations:** ^1^Department of Anesthesiology & Pain Medicine, University of Washington, 1959 NE Pacific Street, Box 356540, Seattle, WA 98195-6540, USA; ^2^Department of Anesthesia, Critical Care and Pain Medicine, Massachusetts General Hospital, Boston, MA, USA; ^3^International Spine, Pain and Performance Center, Washington, DC, USA; ^4^Department of Anesthesiology, Perioperative and Pain Medicine, Stanford University, Palo Alto, CA, USA

## Abstract

**Purpose:**

Wide variability persists in the preparation and storage of common anesthetic medications despite the recognition of anesthesia workspace standardization as a national quality improvement priority. Syringe contamination and medication swaps continue to pose significant hazards to patient safety.

**Methods:**

We assessed differences in practice related to the availability of commonly prepared anesthetic medications. Using baseline provider surveys (*n* = 87) and anesthesia workspace audits (*n* = 80), we designed a custom syringe organization device using 3D printing techniques to serve as a cognitive aid and organizational tool. We iteratively tested and then deployed this device in all 60 operating rooms at a single institution, and then, repeated postintervention surveys (*n* = 79) and workspace audits (*n* = 75) one year after introduction.

**Results:**

Implementation was associated with significant improvements in provider-reported medication availability during coverage and handoff situations (43.7% versus 76.2% reporting 95% confidence preintervention versus postintervention, *p* < 0.001). This was substantiated by audits of the anesthesia workspace which demonstrated reduced variability in the location (*p* < 0.001) and availability (*p* < 0.001) of key medications. Provider confidence in the cleanliness of syringes was also improved (*p*=0.01). A high degree of acceptance and compliance with the intervention was reported, with 80.4% of syringes observed to be stored in the device one year after implementation and approximately 95% of respondents reporting positive measures of usability and convenience.

**Conclusion:**

Use of a simple organizational device for syringes in the anesthesia workspace has numerous safety benefits. 3D printing offers improvements in adaptability and affordability compared with prior approaches.

## 1. Introduction

The availability of key medications in the anesthesia workspace (e.g., succinylcholine, propofol, and vasopressors) is essential to patient safety in the operating room (OR) [1–4]. However, significant interprovider variability exists in the preparation and storage of these medications, even within institutions [5, 6]. The extent of this practice variation poses several potential hazards, particularly in supervision and care team models and in settings with frequent intraoperative handoffs. These hazards include delayed responses to critical changes in patient status, syringe swaps, environmental contamination of syringes [7, 8], and cross-contamination of syringes between patients [9].

Standardization in the preparation of injectable medications has been proposed as a method of reducing these risks [10–12]. However, despite the increasing use of both prefilled syringes and automated labeling systems, medication errors and syringe contamination persist as widespread challenges to the safe delivery of anesthesia [13]. In current anesthesia practice, roughly one in fifteen syringes is estimated to be contaminated with culturable quantities of potentially pathogenic bacteria [7, 8, 14]. Because of the use of multiple syringes in each case, the rate of intravenous (IV) system (i.e., stopcock and tubing inner lumen) contamination is estimated at approximately one in six [8, 14–16]. A growing body of evidence had linked postoperative healthcare-associated infections to such microorganisms within the anesthesia workspace, prompting the recent release of the Society for Healthcare Epidemiology of America's first infection prevention guidelines for the anesthesia work area [17]. This expert guidance document acknowledges many of the challenges to providing effective anesthesia care while maintaining standards for environmental cleanliness and medication sterility. This inherent conflict highlights the need to reengineer the anesthesia environment to facilitate effective infection prevention and medication safety practices, rather than simply mandating behavioral change.

We report the results of a study aimed at improving the handling, availability, and standardization of key medications in the anesthesia workspace, including paired surveys and observational audits before and after deployment of a novel user-designed device for organizing medication syringes. This open-source, 3D-printable syringe bracket system is intended to provide (1) a cognitive visual aid which may reduce the risk of syringe swaps and improve standard medication availability and (2) organizational features to reduce syringe contamination by other items in the anesthesia workspace and the risk of cross-contamination between cases.

## 2. Materials and Methods

As part of a multiphase quality improvement initiative, we (1) assessed baseline provider differences in the preparation and storage of key anesthetic medications in routine practice, (2) developed a novel device to standardize the organization of these medications based on survey data and end-user feedback, and (3) evaluated provider practices and perspectives on use of the device following hospital-wide implementation. This project was undertaken as a quality improvement initiative at Massachusetts General Hospital, and as such was not formally reviewed by the IRB per institutional policy.

### 2.1. Baseline Assessment

We performed a baseline survey (April–September 2015) of perceptions and practices related to the availability, storage, and safety of commonly prepared injectable medications. Using an iPad-based, distributed data collection tool (FileMaker Go v. 14, FileMaker Pro v. 13, Santa Clara, CA, USA), peer surveyors performed convenience sampling of attending anesthesiologists, CRNAs, and residents during the maintenance phase of routine anesthetics. All noncardiac operating room schedules were reviewed on study days and visited at random. Providers were eligible to be surveyed only once, and an encoded version of the employee identification barcode (scanned prior to survey administration) was used to prevent multiple responses by the same individual. Surveyors monitored the case during survey administration to allow the primary anesthetist to self-complete the assessment. Responses were concealed by a confirmation screen upon completion and were not visible to the surveyor upon return of the handheld device. Missing data were minimized through use of electronic validation checks at the time of response entry. Survey questions (Supplementary Table 1) addressed are as follows: (1) the perceived availability of key medications during intraoperative coverage and handoff periods, (2) syringe storage practices, (3) confidence in the cleanliness (not contaminated by the environment or by exposure to a prior patient) of syringes prepared by other providers, and (4) the recalled frequency of safety incidents related to the availability of these medications.

At the time of survey administration, a simultaneous observational audit of medication preparation practices was performed. The availability and location of phenylephrine, ephedrine, succinylcholine, propofol, and glycopyrrolate were recorded by the surveyor.

### 2.2. Drug Bracket Development

**Figure 1:**
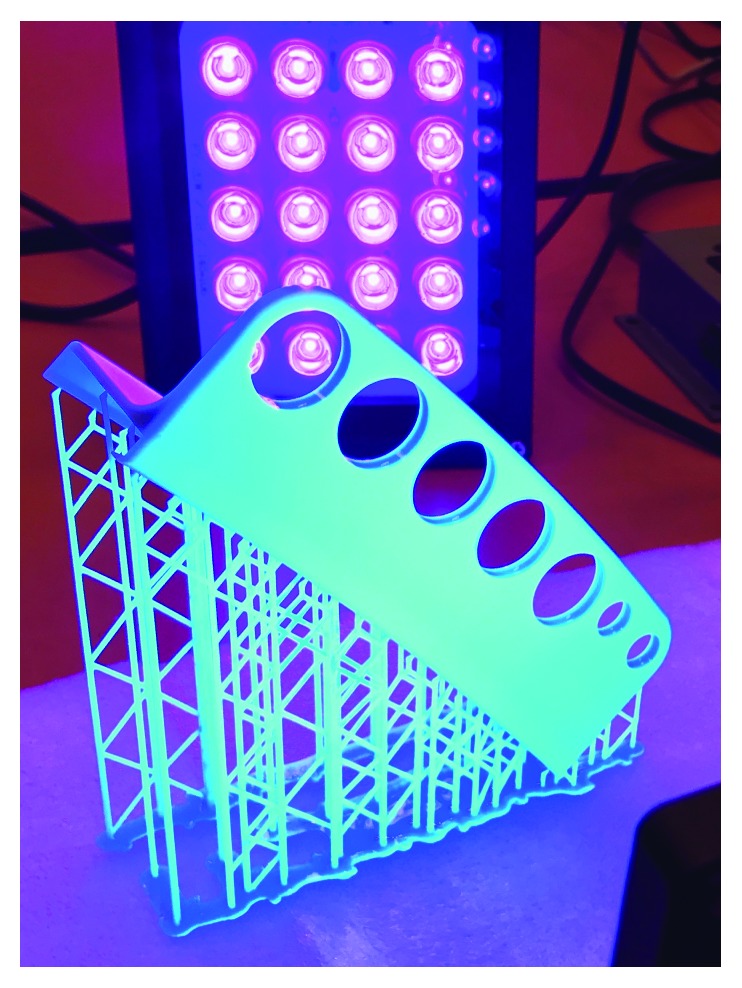
Prototype syringe bracket with removable support structures printed with a desktop stereolithography 3D printer.

**Figure 2:**
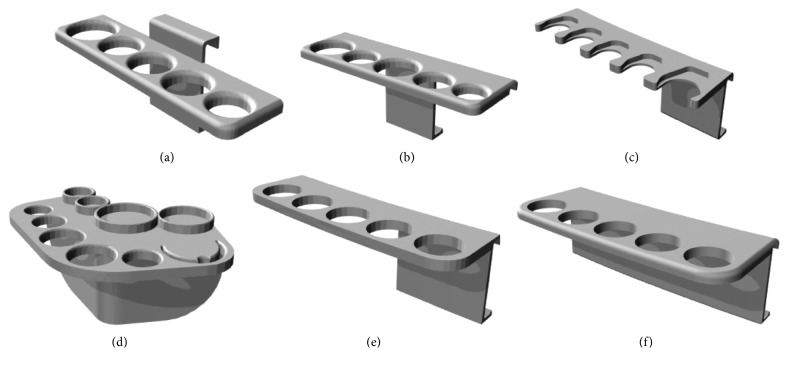
Serial syringe bracket designs based on iterative prototyping and user feedback: (a) initial prototype, (b) elevation of main surface to provide further clearance from anesthesia machine display, (c) alternative slot configuration using flange to hold syringe and allow front-loading and unloading, (d) corner-mounted design including holders for unopened medication vials and a bougie, (e) anterior extension of main surface to provide further clearance from machines with mounted depth of anesthesia monitors, (f) final design with wider support clip for increased stability. A detailed review of the rationale and utility of each of these design features is provided in Supplementary Table 2.

Based on data from the baseline assessment, we identified interprovider variability in the organization of common injectable medications in the anesthesia workspace as an opportunity for quality improvement. Using computer-aided design software free to the academic community (AutoCAD and Fusion 360, Autodesk Inc., San Rafael, CA, USA), we developed a customized bracket designed to store and display five key medications in a convenient, standardized location in the anesthesia workspace. Serial prototypes were manufactured using a desktop stereolithography 3D printer (Formlabs Form 2, Somerville, MA, USA) (Figure 1), tested in the clinical environment, and adapted based on feedback from stakeholders in an iterative fashion (Figure 2). Examples of changes made based on user feedback include mounting location on anesthesia machine, material color, material finish, and elimination of 90-degree angles to facilitate cleaning between cases, modifications in the size and horizontal separation of syringe holes, offset from depth of anesthesia displays, elimination of recesses for unopened medication vials, and separation of the bougie holder (using an independent attachment in a more customary location). The rationale and ultimate utility of each of these design changes are further detailed in Supplementary Table 2.

**Figure 3:**
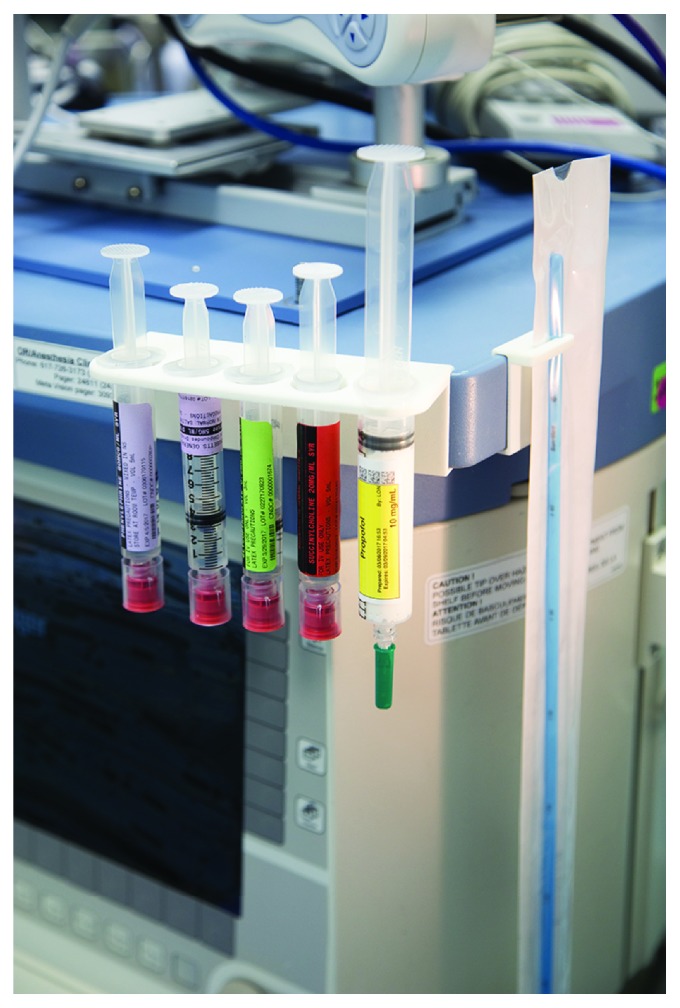
Final selective laser-sintering 3D-printed bracket and accompanying bougie holder in use. The bracket clips securely to the corner of the anesthesia machine and accepts five 10–20 mL BD syringes (standard setup including phenylephrine, ephedrine, glycopyrrolate, succinylcholine, and propofol shown).

The final clip-on device (Figure 3, CAD files included in Electronic Supplementary Material) was produced using a commercial selective laser sintering service (Shapeways, New York, NY). An alternative version of the device accommodating a larger number of smaller syringes was developed for use in pediatric ORs. Implementation was advertised at departmental conferences, on digital announcement displays, by email, and in common lounge spaces. To prevent cross-contamination of syringes between cases and environmental contamination of syringe contents within cases, providers were instructed to store only capped, unused syringes in the bracket. In this unidirectional workflow, once a syringe is removed from the bracket and placed in the “active area” of the anesthesia workspace (machine tray) for patient use, it should not be returned to the “clean area” of the anesthesia workspace (bracket and back supply/medication cart). The empty slot is then immediately restocked by the provider with a clean, unused syringe of the same type for future use. Brackets were installed in the same location on Apollo anesthesia machines (Dräger, Lübeck, Germany) in all 60 noncardiac ORs, including pediatric and obstetric locations (July 2016). A parallel set of brackets for Fabius Tiro and Fabius MRI machines was separately developed but not included as part of this study.

### 2.3. Postimplementation Assessment and Survey

One year after deployment (May–August 2017), a postimplementation assessment was performed including all elements from the baseline survey as well as new questions related to use of the medication bracket.

### 2.4. Statistical Analysis

Data were compiled in a MySQL database (Oracle Corporation, Redwood Shores, CA, USA) and analyzed using R v. 3.4.1 (R Foundation for Statistical Computing, Vienna, Austria). Preintervention and postintervention values were compared using chi-square and Mann–Whitney tests for categorical and ordinal data, respectively. Given the exploratory nature of the project, a power calculation was not used to determine sample size. Rather, we aimed to include the majority of providers routinely providing general anesthetic care in our department (or approximately 80 subjects preintervention and 80 subjects postintervention). At this size, we estimated 80% power to detect a difference of approximately 0.33 points or more on a five-point survey response scale.

## 3. Results

A total of 87 baseline surveys and 80 observational audits were completed. One year after device deployment, 79 follow-up surveys and 75 observational audits were completed for comparison. Attending anesthesiologists (31% pre, 35% post), nurse anesthetists (31% pre, 32.5% post), and residents/fellows (37.9% pre, 32.5% post) were similarly represented and the distribution of respondents by role did not differ significantly between phases (*p*=0.75, Table 1 (A)).

**Figure 4:**
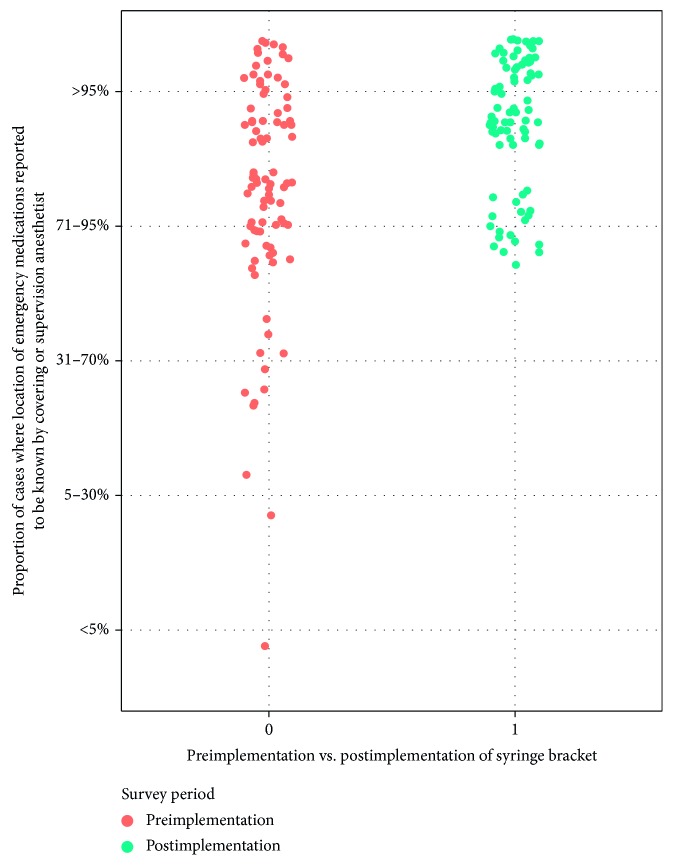
Provider responses to the following question, before versus after hospital-wide deployment of the emergency medication bracket: “In thinking about the last three months, when you have taken over a case from someone else OR are supervising. Another provider, approximately what percentage of the time do you know where the emergency drugs (phenylephrine, ephedrine, propofol, succinylcholine, glycopyrrolate) are located?” Self-reported confidence was increased from a mean of 4.25 to 4.76 on 5-point scale, preimplementation vs postimplementation (*p* < 0.001).

In the subjective component of the survey, providers reported significantly higher levels of confidence in knowing the location of emergency medications when supervising or taking over cases during the prior three months (*p* < 0.001, Table 1(B)). Following device deployment, 76.2% of respondents reported greater than 95% confidence in knowing the location of these medications during supervision or handoffs, compared with 43.7% at baseline (Figure 4). Respondents also reported being more confident that emergency medications prepared by other providers were clean (not exposed to a prior patient or contaminated by the environment) (*p*=0.01, Table 1 (C)). There was no change in the recalled frequency of incidents related to the availability of emergency medications (*p*=0.47, Table 1 (D)).

In the objective component of the survey, we observed reduced variability in the location of emergency medications within the anesthesia workspace (*p* < 0.001, Table 1 (E)) and a high degree of device acceptance (80.4% of syringes not actively in use were observed to be stored in the bracket). Bracket implementation was also associated with an increase in the observed availability of several medications (Table 1 (F)).

Provider feedback on device usability was generally positive. One year after deployment, 94% of users reported that they found the device to be helpful, 96.3% expressed a desire to have the brackets expanded to nonoperating room anesthetizing locations, and 96.2% would like to have them in other hospitals where they may work at present or in the future. The most cited benefits of the device were convenience (84%), ease in locating emergency medications (81%), practice standardization (76%), cleanliness (73%), and improved perception of safety (50%). The most common requests for improvement and modification included number of syringe holes (13%), size of syringe holes (10%), location (6%), appearance (3%), and requests for other features (3%).

## 4. Discussion

In a clinical environment in which injectable medication preparation and labeling systems are increasingly standardized, we identified variability in the location of medications stored within the anesthesia workspace as an important additional contributor to the cleanliness and availability of key injectable medications. Using affordable 3D printing techniques, we developed a series of customized medication brackets which can be clipped noninvasively to the anesthesia machine, easily cleaned between cases, and exchanged to suit the nature of the anesthetic being performed (e.g., adult versus pediatric). Implementation of the device was associated with improvements in both domains of syringe cleanliness and essential medication availability.

Prior studies have demonstrated marked individual and regional variation in practices related to the cleanliness of injectable medications prepared by anesthesiologists. A recently published survey of members of the Canadian Anesthesiologists' Society reported that practitioners continue to knowingly share medication vials (83%), syringes (7%), and needles (2%) between multiple patients [18]. The most frequently cited reasons for these practices were the desire to reduce cost and waste. Similar values have been reported in a survey of anesthesiologists in New York state, who further cited medication shortages as a reason for sharing used medication vials between patients [19]. While these surveys reflect the prevalence of medication practices known to increase the risk for infection transmission, practitioners may be unaware of many environmental and cross-contamination events. Sampling of syringes and IV systems used in actual anesthetic care has demonstrated contamination rates of 2–17% [7, 8, 14] and 6–32%, [8, 14–16] respectively, in real-world practice. The bacteria isolated in these studies include an appreciable number of Gram-negative organisms and suggest the possibility that contact with other items within the anesthesia workspace serves as a source of contamination. The significance of contact between syringes and unclean items in the anesthesia workspace is further supported by detailed observations in simulated operating environments, showing a high rate to contact between syringes in active use with other items collocated on the surface of the anesthesia workspace and association with syringe contamination [14].

Use of a dedicated syringe bracket such as the device used in our study has the potential to reduce syringe-associated transmission events in two ways. First, it may reduce the risk of environmental contamination by providing a clean location for syringe placement, physically separating these medications from items such as airway equipment, vascular access devices, and monitoring equipment. Second, it may prevent cross-contamination between patients by clearly distinguishing unused, preprepared emergency medications from those that have been accessed for a given case (in this study, we promoted use of a “one-way” system in which syringes were advanced from the medication drawer to the bracket to the active workspace and never placed back in the bracket once accessed for use). As a by-product, this distinction may help prevent medication waste by reducing the frequency with which clean medications are discarded due to uncertainty about potential contamination. In our survey, 28% of respondents reported occasionally discarding and replacing emergency medications for this reason at baseline, versus 14% following implementation of the medication bracket.

In addition to infection transmission, medication errors related to the use and storage of preprepared medication in the anesthesia workspace pose a significant threat to patient safety. Medication errors are the single most common cause of malpractice cases against anesthesiologists in Canada and account for approximately two-thirds of all damages awarded [20]. Subsequent efforts to standardize labeling and organization of medication carts [21] address the risk of mistaken or mis-stocked medication vials. However, in a survey of Canadian Anesthesiologists' Society members, the majority of medication errors were attributed to “syringe swaps” (60%), rather than in misidentification (39%) or mis-stocking (18%) of ampoules/vials [22]. Standardized labeling and scan-before-administration barcoding systems have been proposed as a method of addressing syringe swaps [10]. However, barriers related to cost, efficiency, and integration with existing technology have limited the widespread implementation of real-time medication scanning in the operating room. Further, even in hospital systems such as ours where the use of automated medication labeling systems is routine, the problem of syringe substitution errors has persisted, involving not only “rare use” or “high-risk” medications but frequently resulting from “swaps” of the most commonly used anesthetic medications [13].

To address these challenges, a variety of medication trays, bins, and organization systems have been described [23–25]. These solutions similarly provide a standardized location and arrangement for predrawn medication syringes, as well as a visual cue as to any item that may be missing. Although the benefits of these organization devices have repeatedly been demonstrated in the institutions from whence they are derived, more widespread adoption has been hindered, perhaps by (1) the lack of customizability (different institutions and various anesthetic case types may have widely different requirements in terms of syringe type and number) and (2) the lack of a commercial product meeting this need due to the simplicity of the solution and perceived lack of profitability. In comparison with prior approaches, the utilization of 3D printing technologies may provide a more customizable and cost-effective approach to implementation. Using this method, we developed a series of customized syringe bracket designs that can be rapidly adapted based on institutional needs, can be modified to suit various case types (e.g., adult versus pediatric and variations in syringe size and number), and can be produced with and deployed at low cost.

The limitations of this report include the lack of a control group inherent in the pre-post-implementation study design and the potential for confounding by concurrent changes in practice. No shortage of any medication referenced in the survey or audit instrument occurred during the study period and no concurrent quality improvement or educational efforts related to medication safety were conducted during this time. The same syringe types, medication carts, and labeling system (Safe Label System, Codonics, Cleveland, OH, United States) also remained in use throughout this period. Important considerations related to the generalizability of this solution include local requirements for medication storage between cases and the compliance of nonessential equipment in operating areas with standards for sanitization, as further discussed in the Electronic Supplementary Material.

## 5. Conclusions

In summary, in a single-center quantitative quality improvement initiative utilizing a preintervention and postintervention design, we observed improvements in the perceived cleanliness and availability of common injectable medications following implementation of a novel organization device. Measures of practitioner adoption and satisfaction with the device one year after implementation suggest that this intervention resulted in a high-value, meaningful culture change and may yield similar improvements outside of our own institution. Given the potential interest in adapting this design for use in other practice settings and with various machine types, we have included the original CAD files and design templates for printing or design adaptation in the Electronic Supplementary Material and as well as the NIH 3D Print Exchange platform in an open-source fashion. [26].

## Figures and Tables

**Table 1:** Comparison of participant composition by clinical role, self-reported survey responses, and results of simultaneous observational audit, before and after implementation of system-wide implementation of emergency medication syringe brackets. Values reported as count (percentage).

	Preimplementation (*n* = 87)	Postimplementation (*n* = 80)	*p* value
Demographics			
A. Provider roles			
Attending	27 (31.0)	28 (35.0)	0.75
CRNA	27 (31.0)	26 (32.5)	
Resident/fellow	33 (37.9)	26 (32.5)	
Survey responses			
B. Know location of emergency medications			
<5%	1 (1.1)	0 (0.0)	<0.001
5–30%	2 (2.3)	0 (0.0)	
31–70%	9 (10.3)	0 (0.0)	
71–95%	37 (42.5)	19 (23.8)	
>95%	38 (43.7)	61 (76.2)	
C. Confidence in cleanliness of emergency medication syringes			
I am rarely ever sure about this	5 (5.7)	2 (2.5)	0.01
Now and then I have to draw one up because I am uncertain	24 (27.6)	11 (13.8)	
With extremely rare exception, I am confident	47 (54.0)	50 (62.5)	
I am always 100% certain they are clean	11 (12.6)	17 (21.3)	
D. Recalled incidents in which emergency medication unavailable			
None	40 (46.0)	40 (50.0)	0.47
1-2	32 (36.8)	31 (38.8)	
3–5	13 (14.9)	5 (6.3)	
More than 5	2 (2.3)	4 (5.0)	
Observational audit			
E. Location of individual emergency medication syringes			
Medication bracket	0 (0)	271 (74.0)	<0.001
Top surface of anesthesia machine	201 (38.4)	8 (2.2)	
Anesthesia machine tray	84 (16.1)	51 (13.9)	
Surface of omnicell	200 (38.2)	7 (1.9)	
In use or connected to stopcock	38 (7.3)	29 (7.9)	
F. Actual medication availability			
Phenylephrine	80 (92)	76 (95)	0.43
Propofol	81 (93)	74 (93)	0.88
Ephedrine	74 (85)	76 (95)	0.03
Succinylcholine	53 (61)	71 (89)	<0.001
Glycopyrrolate	53 (61)	72 (90)	<0.001

## Data Availability

Original STL printing files and CAD templates in Fusion 360 format are included in the Supplementary Material. A version of the adult bracket adapted for the Dräger Apollo anesthesia machine is also available in the NIH 3D Print Exchange Library (Model ID 3DPX-009876) at: https://3dprint.nih.gov/discover/3dpx-009876.

## Supplementary Materials

Supplementary Materials Supplementary Table 1: Preintervention and postintervention survey questions, extracted from tablet-based survey instrument. Supplementary Table 2: Rationale for iterative changes to bracket design as shown in Figure 2. File: “syringe bracket template.f3d” Description: Original Fusion 360 design file including timeline-based design history. Allows for easy modification of hole size, number, shelf width, and other parameters. File: “syringe bracket adult.stl” Description: Adult version of STL file used for printing. Allows for simple viewing and direct 3D printing without design modification. File: syringe bracket pediatric.stl” Description: Pediatric version of STL file used for printing. Allows for simple viewing and direct 3D printing without design modification.
