# Supplementary material for: Anesthesia Workspace Cleanliness and Safety: Implementation of a Novel Syringe Bracket Using 3D Printing Techniques
Source: Anesthesiol Res Pract. 2019 Jul 1;2019:2673781. doi: 10.1155/2019/2673781 (PMC6636519; doi:10.1155/2019/2673781)
Supplement: Supplementary Materials — Supplementary Table 1: Preintervention and postintervention survey questions, extracted from tablet-based survey instrument. Supplementary Table 2: Rationale for iterative changes to bracket design as shown in Figure 2. File: “syringe bracket template.f3d” Description: Original Fusion 360 design file including timeline-based design history. Allows for easy modification of hole size, number, shelf width, and other parameters. File: “syringe bracket adult.stl” Description: Adult version of STL file used for printing. Allows for simple viewing and direct 3D printing without design modification. File: syringe bracket pediatric.stl” Description: Pediatric version of STL file used for printing. Allows for simple viewing and direct 3D printing without design modification. [file 2673781.f1.zip › 2673781.f1/mat.2673781.v2.pdf]

Supplementary Table 1: Pre- and post-intervention survey questions, extracted from tablet-based survey instrument.

Pre-intervention Survey	Post-intervention Survey
Stem: In thinking about the last three months, when you have taken over a case from someone else OR are supervising another provider:	Stem: In thinking about the last three months, when you have taken over a case from someone else OR are supervising another provider:
**1. Approximately what percentage of the time do you know where the emergency drugs (phenylephrine, ephedrine, propofol, succinylcholine, glycopyrrolate) are located?**<br><5% 5-30% 31-70% 71-95% >95%	**1. Approximately what percentage of the time do you know where the emergency drugs (phenylephrine, ephedrine, propofol, succinylcholine, glycopyrrolate) are located?**<br><5% 5-30% 31-70% 71-95% >95%
**2. Approximately what percentage of the time are each of these emergency drugs immediately available?**<br>2-A. Phenylephrine<br><5% 5-30% 31-70% 71-95% >95%<br>2-B. Ephedrine<br><5% 5-30% 31-70% 71-95% >95%<br>2-C. Propofol<br><5% 5-30% 31-70% 71-95% >95%<br>2-D. Succinylcholine<br><5% 5-30% 31-70% 71-95% >95%<br>2-E. Glycopyrrolate<br><5% 5-30% 31-70% 71-95% >95%	**2. Approximately what percentage of the time are each of these emergency drugs immediately available?**<br>2-A. Phenylephrine<br><5% 5-30% 31-70% 71-95% >95%<br>2-B. Ephedrine<br><5% 5-30% 31-70% 71-95% >95%<br>2-C. Propofol<br><5% 5-30% 31-70% 71-95% >95%<br>2-D. Succinylcholine<br><5% 5-30% 31-70% 71-95% >95%<br>2-E. Glycopyrrolate<br><5% 5-30% 31-70% 71-95% >95%
**3. How confident are you that the emergency drugs on the anesthesia machine or back cart are clean (not contaminated from prior case)?**<br>I am rarely ever sure about this<br>Now and then I have to draw one up because I am uncertain<br>With extremely rare exception, I am confident they are clean<br>I am always 100% confident they are clean	**3. How confident are you that the emergency drugs on the anesthesia machine or back cart are clean (not contaminated from prior case)?**<br>I am rarely ever sure about this<br>Now and then I have to draw one up because I am uncertain<br>With extremely rare exception, I am confident they are clean<br>I am always 100% confident they are clean
**4. Where do you think the ideal location is for keeping clean emergency drugs?**<br>On top of the anesthesia tray<br>On top of the anesthesia machine (by vital sign monitor)<br>Otherwise attached to the anesthesia machine<br>On top of the Omnicell workstation<br>Other (free text entry)	**4. Where do you think the ideal location is for keeping clean emergency drugs?**<br>On top of the anesthesia tray<br>On top of the anesthesia machine (by vital sign monitor)<br>Otherwise attached to the anesthesia machine<br>On top of the Omnicell workstation<br>Syringe bracket<br>Other (free text entry)
**5-A. In the last three months, how many instances have there been in which you wish one of the five emergency drugs listed above had been more immediately available?**<br>None<br>1-2 times<br>3-5 times<br>More than 5 times	**5-A. In the last three months, how many instances have there been in which you wish one of the five emergency drugs listed above had been more immediately available?**<br>None<br>1-2 times<br>3-5 times<br>More than 5 times
**5-B. If thinking about specific instances, which drug(s) do you wish had been more readily available?**<br>(free text entry)	
	**6. In your opinion, has the addition of the emergency drug brackets been useful?**<br>Yes No
	**7. What what are the best features of the device? (check all that apply)**<br>convenience<br>ease of knowing where emergency drugs are located<br>practice standardization<br>workspace cleanliness<br>improved safety<br>none of the above
	**8. In regard to the drug bracket, which of these are problematic or need improvement? (check all that apply)**<br>location<br>number of holes<br>size of holes<br>appearance<br>Other (free text entry)
	**9. Based on your experience, would this be helpful to have in other locations at MGH (e.g. iMRI, endoscopy, radioloy, EP)?**<br>Yes No
	**10. Imagining working at another hospital as part of a future job or rotation, would you like to have a similar drug bracket in your OR?**<br>Yes No

Supplementary Table 2: Rationale for iterative changes to bracket design as shown in Figure 2.

Bracket Version	Changes
A	(Original design)
B	Bracket surface on which syringe flanges rest elevated to create more distance between inferior extent of syringes and vaporizers to maintain normal access to vaporizers. This design change was carried forward to all subsequent versions.
C	Recess for syringe insertion changed from simple holes to impression matching syringe flanges. This design change was intented to allow syringes to be lifted outward instead of upward (convenience feature) and to enforce use of a standardized syringe arrangement and orientation. However, in this configuration, the syringes were more prone to being dislodged when reaching for the nearby depth of anesthesia monitor and the design was not forward-compatible with changes to syringe size and brand. The clip attaching the bracket to the machine was shifted to the far right of the device to bring the side of the bracket flush with the right side of the anesthesia machine and prevent damage during machine movement and room turnover. This design change was carried forward to versions E and F.
D	The mounting position of the bracket was changed to wrap around the right corner of the anesthesia machine. This provided additional surface area for inclusion of rings for holding unopened medication vials (mimicing baseline practice). The corner mounting position made attachement and replacement of the device (for cleaning, replacement, etc.) more difficult and was not carried forward. The rings for holding empty vials were effective, but created acute angles which were difficult to wipe for decontamination between cases and were not forward-compatible with changes in vial size. The addition of a bougie holder was a popular feature, but was carried forward to subsequent designs as a separate attachment (placed on the right lateral side of the anesthesia machine in a customary location consistent with baseline practice).
E	Holes for syringe insertion were changed to a standard intermediate diameter able to securely accomodate both 10 and 20 mL syringes. This change was carried forward to version F.
F	(Final design) The mounting clip was enlarged horizontally to create a more secure attachment to the lip of the anesthesia machine. Ninety degree angles were smoothed with a fillet function to optimize effective wiping for decontamination between cases.

Emergency Medication Bracket, CAD Files

The following items are included as separate Supplemental Digital Content files:

Fusion 360 Archive format

File: "syringe bracket template.f3d"

Description: Original Fusion 360 design file including timeline-based design history. Allows for easy modification of hole size, number, shelf width, and other parameters.

STL format

File: "syringe bracket adult.stl"

Description: Adult version of STL file used for printing. Allows for simple viewing and direct 3D printing without design modification.

File: "syringe bracket pediatric.stl"

Description: Pediatric version of STL file used for printing. Allows for simple viewing and direct 3D printing without design modification.

Considerations related to infection control

The ASA Committee on Occupational Health Task Force on Infection Control has provided the following practical primer on infection control related to the cleaning of non-critical devices and environmental surfaces in the operating room (see section I):

"Recommendations for Infection Control for the Practice of Anesthesiology (Third Edition)"
https://www.asahq.org/resources/resources-from-asa-committees#ic

Both design features (e.g. use of filleted features rather than acute internal angles) and material considerations (e.g. use of printing and finishing techniques which produce smooth, wipeable surfaces) may impact the ability of 3D printed materials to be cleaned and the acceptability of such objects is subject to local, state, and hospital policy.
